# News and Coming Events

**Published:** 1907-09-14

**Authors:** 


### NEWS AND COMING EVENTS.

The inaugural address of the winter session of the London School of Tropical Medicine will be delivered on Monday, October 21, by Sir T. Lauder Brunton, M.D.

---

The Sixth International Dermatological Congress is being held at the Academy of Medicine, New York. The opening meeting took place on September 9, under the presidency of Dr. James C. White, of Boston.

---

The congratulations of the medical profession are due to Sir Henry Pitman on the attainment of his hundredth birthday. Sir Henry, whose reputation is naturally less known among the younger generation of practitioner, is senior consulting physician to St. George's Hospital, and must be the doyen of the consulting and visiting staffs of the London hospitals.

---

The winter session of St. Bartholomew's Hospital and Medical College begins on October 1 next. Six entrance scholarships of a total value of £420 will be competed for on September 23 next. For the full regulations regarding the scholarship examinations and the various courses, application should be made to the Dean, St. Bartholomew's Hospital, E.C.

The death of Mr. Timothy Holmes, F.R.C.S., on the 8th inst. at the age of eighty-three, removes from our midst one of the most notable survivors of the older school of surgeons; those we mean who had established reputations before the introduction of antiseptic methods. As student, house surgeon, registrar, surgeon, consulting surgeon, and treasurer, Mr. Holmes's connection with St. George's Hospital dates back to 1847, in which year he left Cambridge to study there. For many years his reputation among students as an exacting examiner in anatomy and surgery stood almost higher than among the public and the profession as the type of all that a consultant should be. To intellectual gifts of a very high order, Mr. Holmes united a degree of tact in dealing with patients and of painstaking care in diagnosis which ensured a well-filled consulting-room. The misfortune of the loss of an eye, and the revolution in surgical procedures consequent on the antiseptic regime, somewhat dimmed his reputation as an operator in his later years, but as a diagnostician his powers were always held in the highest esteen. Besides his "System of Surgery," to which he contributed largely himself, the deceased surgeon edited "Gray's Anatomy," and wrote a life of Sir Benjamin Brodie. He was for many years chief surgeon to the Metropolitan Police Force.

The Committee of the Liverpool Workhouse has decided to sanction the appointment of a resident medical superintendent for the Highfield Infirmary at an annual salary of £200, together with the usual allowances.

According to an American contemporary, the Sunday regulations in an Arkansas town prohibit the sale of ice on that day, unless on an order of a local physician, who is, moreover, required to write a regular prescription for the commodity.

The Second International Congress of Physiotherapy will this year be held in Rome, commencing on Oct. 13 next. Special travelling facilities have been granted to practitioners attending the Congress by the Italian Ministry of Public Works. The General Secretary of the Congress is Professor Carlo Colombo, Via Plinio, Rome.

The Countess of Aberdeen opened the new wing of the Rotunda Hospital, Dublin, on Aug. 27 last. The addition has cost more than £8,000 to build and furnish, and increases the accommodation of the hospital by six extra wards. It provides nurses' rooms, rooms for students, disinfecting rooms, and a well-arranged series of bathrooms.

The newly erected bonded warehouse of Messrs. James Buchanan and Co., the well-known whisky distillers and blenders, is the largest building of its kind in the world. The warehouse has a capacity of 20,000 butts, representing a duty of more than a million and a half pounds sterling. The capacity of the eleven blending vats is more than 130,000 gallons. The warehouse, which is situate in Washington Street, Glasgow, was recently opened.

It has been decided to take steps to provide Bargoed and New Tredegar with a joint cottage hospital. The initiative in the matter was taken by the Powell Dufryn Collieries Company, whose directors offered to erect and equip a cottage hospital and to hand the same over to the workmen of the company upon condition that the maintenance and upkeep would be provided for by the men. At a mass meeting of the employés held recently at Bargoed it was unanimously decided to accept the offer of the company.

The subject for the prize essay in terms of the bequest of the late Dr. John Parkin, who left a sum of money in trust to the Royal College of Physicians of Edinburgh to found an annual prize for the best essay on certain subjects connected with medicine, is this year "The curative effects of carbonic-acid gas or other forms of carbon in cholera, for different forms of fever, and in other diseases." The value of the prize is £100, and essays, which must comply with the usual equipments, are to be in the hands of the Secretary of the Royal College of Physicians not later than December 31, 1907.

In the current issue of "The World's Work" (William Heinemann, 1s.) Mr. Edgar Forbes discusses the question "Is the Doctor a Shylock?" After reviewing the whole matter, for and against, he enters a strong "protest against careless statements, growing out of exceptional experiences, which reflect on the medical profession at large—a profession which does more practical relief work without any reward than any other class of men." He declares, after a consideration of statistics, that "something like 20 per cent. of physicians in the larger towns have handsome incomes, in return for expert work, and much of it; 80 per cent. of city physicians and most country doctors make little more than a decent living." The article is well written, and will repay perusal.

The Chelsea Hospital for Women has received a bequest of £1,000 under the will of the late Mrs. Atkinson Bolger.

The selling agents in Great Britain for Horsford's acid phosphate are Bovril, Limited, 152-166 Old Street, London, E.C.

Miss Helen Mayhurst has forwarded £50, being the annual instalment for the endowment of a "St. Martin Bed," to the Secretary of the Lady Margaret Fruitarian Hospital, Bromley.

A new cure for alcoholism has been discovered in the waters of the Potter Springs, Mingo Junction, Ohio. The local municipality has had the water analysed, and is now selling it to those who believe in its "curative" properties.

The annual distribution of prizes at the Charing Cross Medical School will take place on Tuesday, October 1 next. The prizes will be distributed to the successful students by the Right Hon. the Earl of Kilmorey at four o'clock in the afternoon.

According to the *Atlanta Journal-Record of Medicine*, the United States now consumes five times more opiates than it did six years ago, the average being a drachm for each inhabitant per year. China only takes 27 grains per inhabitant, and European countries only 12 grains.

Where to go for the summer holidays is a question which some holiday makers find difficult to answer. The excellent little handbooks "Summer Holidays" and "A Tourist Guide to the Continent," recently issued by the Great Eastern Railway Company, give full particulars of the various places of interest served by this great line. Both booklets are copiously illustrated.

The Second Norman Kerr Memorial Lecture, held under the direction of the Society for the Study of Inebriety, will be delivered by Robert Welsh Branthwaite, Esq., M.D., H.M. Inspector under the Inebriates Acts, on Tuesday, October 8, 1907, at 8.30 p.m., in the Hall of the Royal Society of Medicine, 20 Hanover Square, London, W. The subject of the lecture will be "Inebriety; its Causation and Control."

The following is the programme of the Cancer Exhibition at the International Surgical Congress to be held at Brussels in September 1908:—The exhibition of macroscopic or microscopic cancer specimens, which are either instructive from a surgical standpoint or of special interest on account of their localisation or method of dissemination; topographical anatomical specimens or drawings illustrating individual operations for cancer; topographical anatomical preparations of the lymphatic vessels and glands of particular regions; statistical wall-charts illustrating the duration of the treatment of cancerous diseases, the frequency of primary cancer and its metastases in particular organs (based upon clinical or pathological experience), and the spread of cancer in different countries; exact plans of so-called cancer "nests"; family trees of families in which cancer has existed for several generations; plans of cancer institutions and hospitals, their reports of work done, and financial statements; agitations for early treatment of cancerous diseases; appeals to the public; proposals as to the means by which a knowledge of the importance of the early diagnosis of cancer may be brought home to the public without exciting panic. The Secretary of the International Surgical Society, under whose auspices the exhibition is to be held, is Professor Depage, 75 Avenue Louise, Brussels, from whom all further particulars may be obtained.

